# Hippocampal changes in inflammasomes, apoptosis, and MEMRI after radiation-induced brain injury in juvenile rats

**DOI:** 10.1186/s13014-020-01525-3

**Published:** 2020-04-10

**Authors:** Jun Yang, Jingyan Gao, Dan Han, Qinqing Li, Chengde Liao, Jindan Li, Rui Wang, Yueyuan Luo

**Affiliations:** 1 Department of Radiology, The Third Affiliated Hospital of Kunming Medical University, Yunnan Cancer Hospital & Cancer Center, No. 519 Kunzhou Road, Xishan District, Kunming, 650118 Yunnan P.R. China; 2 Department of Medical Imaging, The First Affiliated Hospital of Kunming Medical University, No. 295 Xichang Road, Kunming, 650032 Yunnan PR China; 3 Department of Radiation Oncology, The Third Affiliated Hospital of Kunming Medical University, Yunnan Cancer Hospital & Cancer Center, No. 519 Kunzhou Road, Xishan District, Kunming, 650118 Yunnan P.R. China

**Keywords:** Radiation-induced brain injury, Inflammasomes, Apoptosis, Manganese-enhanced MRI, Hippocampus, Pyroptosis

## Abstract

**Purpose:**

The aim of this study was to characterize changes in hippocampal inflammasomes, pyroptosis and apoptosis in juvenile rats after brain irradiation and to assess whether manganese-enhanced magnetic resonance imaging (MEMRI) reflected those changes.

**Materials and methods:**

Four-week-old male Sprague-Dawley rats received a whole-brain radiation dose of 15 Gy or 25 Gy. Hippocampal inflammasomes and apoptosis were measured using Western blot analysis at 4 days and 8 weeks after irradiation. MEMRI and magnetic resonance spectroscopy (MRS) were performed at the same time points.

**Results:**

Neither the 15 Gy nor 25 Gy group showed changes in the expression of inflammasome proteins absent in melanoma 2 (AIM2), gasdermin-D (GSDMD), nucleotide oligomerization domain-like receptor protein 1 (NLRP1) and NLRP3 at 4 days or 8 weeks after radiation injury (*P* > 0.05). Furthermore, the expression levels of the inflammatory cytokines interleukin-1β (IL-1β) and IL-18 were not significantly different among the groups (*P* > 0.05). The expression levels of cleaved caspase-1 and -3, indicators of apoptosis, were higher in the irradiation groups than in the control group at 4 days post irradiation, especially for caspase-3 (*P* < 0.05), but this increase was slightly attenuated at 8 weeks after radiation injury. Four days post irradiation, the MEMRI signal intensity (SI) in the irradiation groups, especially the 25 Gy group, was significantly lower than that in the control group (*P* < 0.05). Eight weeks after radiation injury, the SI of the 15 Gy group and the 25 Gy group recovered by different degrees, but the SI of the 25 Gy group was still significantly lower than that of the control group (*P* < 0.05). On day 4 post irradiation, the metabolic ratio of N-acetylaspartate (NAA) to creatine (Cr) in the 15 Gy group and 25 Gy group was significantly lower than that in the control group (*P* < 0.05). The NAA/Cr ratio in the 15 Gy group recovered to control levels at 8 weeks (*P* > 0.05), but the NAA/Cr ratio in the 25 Gy group remained significantly lower than that in the control group (*P* < 0.05).

**Conclusion:**

Radiation-induced brain injury is dose-dependently associated with apoptosis but not inflammasomes or pyroptosis, and the change in apoptosis can be detected by MEMRI.

## Introduction

Radiotherapy is increasingly widely used in the clinical treatment of primary brain tumors and metastases and has proven effective. An increasing number of phase III clinical trials have shown that patients can benefit from radiation therapy alone or in combination with other treatments [1, 2]. However, in the process of treatment, radiation inevitably damages normal brain tissue while killing tumor cells. Radiation-induced impairment of cognitive function and memory is a side effect of cranial radiation therapy and can significantly affect the quality of life of patients, especially pediatric brain tumor survivors. Many of these survivors exhibit a long-term decline in neurocognitive function that significantly affects quality of life [3, 4]. Furthermore, adverse events are significantly more common with longer courses of whole-brain radiation therapy [5].

The hippocampus is very important for memory function and is particularly susceptible to radiation. Some hippocampal cells are highly proliferative, and studies have shown that the loss of these cells after radiotherapy can lead to cognitive impairment [6]. However, the mechanisms of brain irradiation damage and changes in cognitive function are not fully understood. Radiation-induced brain injury (RIBI) is believed to lead to cell death, neurogenesis impairment, oxidative stress, vascular injury, demyelination and inflammation [7, 8]. In addition, there is evidence that inflammation may play a role in the observed radiation side effects [9]. Inflammasomes are cytoplasmic multiprotein complexes that regulate inflammatory responses associated with tissue injury and infection [10]. Activation of the inflammatory complex can lead to the secretion of proinflammatory cytokines, such as interleukin (IL)-1β and IL-18, and/or the initiation of pyroptosis, which is a proinflammatory and lytic mode of cell death distinct from apoptosis [11, 12]. The abnormally activated inflammatory response induced by the release of cytokines into the microenvironment is related to many pathologies. Since radiation-induced tissue damage also produces an inflammatory response, the role of inflammasomes and pyroptosis in radiation-induced cell and tissue damage is not clear. Stoecklein et al. demonstrated that inflammasome activation occurs in many immune cell types after radiation exposure [13]. Recent studies have indicated that the activation and expression of inflammasomes such as nucleotide oligomerization domain-like receptor protein 3 (NLRP3) are significantly upregulated in the lung, intestine tissue, oral mucosa and skin after radiation injury [14–16]. Liao et al. demonstrated that RIBI involves pyroptosis, including microglia pyroptosis, which may rely on the activation of the NLRP3 inflammasome [17]. However, due to the complexity of radiation damage, the underlying pathophysiology of radiation-induced changes in inflammasomes in the hippocampus is still poorly understood.

The detection of changes in inflammasomes, pyroptosis and apoptosis before the onset of cognitive disorder may act as an early neuroimaging marker of radiation encephalopathy and could indicate the use of therapeutic interventions such as anti-inflammatory or anti-apoptotic agents. Manganese-enhanced magnetic resonance imaging (MEMRI) based on the strong paramagnetism of Mn^2+^ shows an obvious advantage in the field of neuroimaging. Mn^2+^ is absorbed by excitable cells through voltage-gated calcium channels, and its activity-dependent uptake and trafficking correlate with neuronal function [18, 19]. Furthermore, Mn^2+^ is taken up preferentially in the hippocampus [20]. Therefore, MEMRI may provide a valuable way to quantify neuronal function in brain regions affected by RIBI. Magnetic resonance spectroscopy (MRS) is another imaging method that can be used to detect metabolic changes in the hippocampus in a noninvasive manner [21]. MRS can be used to monitor brain tumor progression and is helpful for identifying radiation necrosis after radiation therapy [22, 23]. In studies of radiation brain damage, MRS has been shown to detect early metabolic changes in normal irradiated brain tissue [24–26].

In this study, we used juvenile rats to establish a RIBI model and evaluate hippocampal changes in inflammasomes, apoptosis, pyroptosis, and MEMRI during the acute and early-delayed stages of brain injury.

## Methods and materials

### Animals

The study was approved by our university’s animal experiment review committee, and all animal experiment procedures were performed in accordance with the guidelines for animal care and use. Experimental studies were carried out using 4-week-old male Sprague-Dawley rats (50–80 g) from the Department of Experimental Animals at our university. Sixty rats were randomized into sham group (0 Gy, *n* = 10 for day 4 and *n* = 10 for 8 weeks), and 2 experimental groups (15 Gy and 25 Gy, *n* = 10 for day 4 and *n* = 10 for 8 weeks each). The rats were housed in clear plastic cages at a room temperature of 23 °C with a 12 h light/dark cycle and allowed free access to water and food.

### Cranial irradiation protocol

**Fig. 1:**
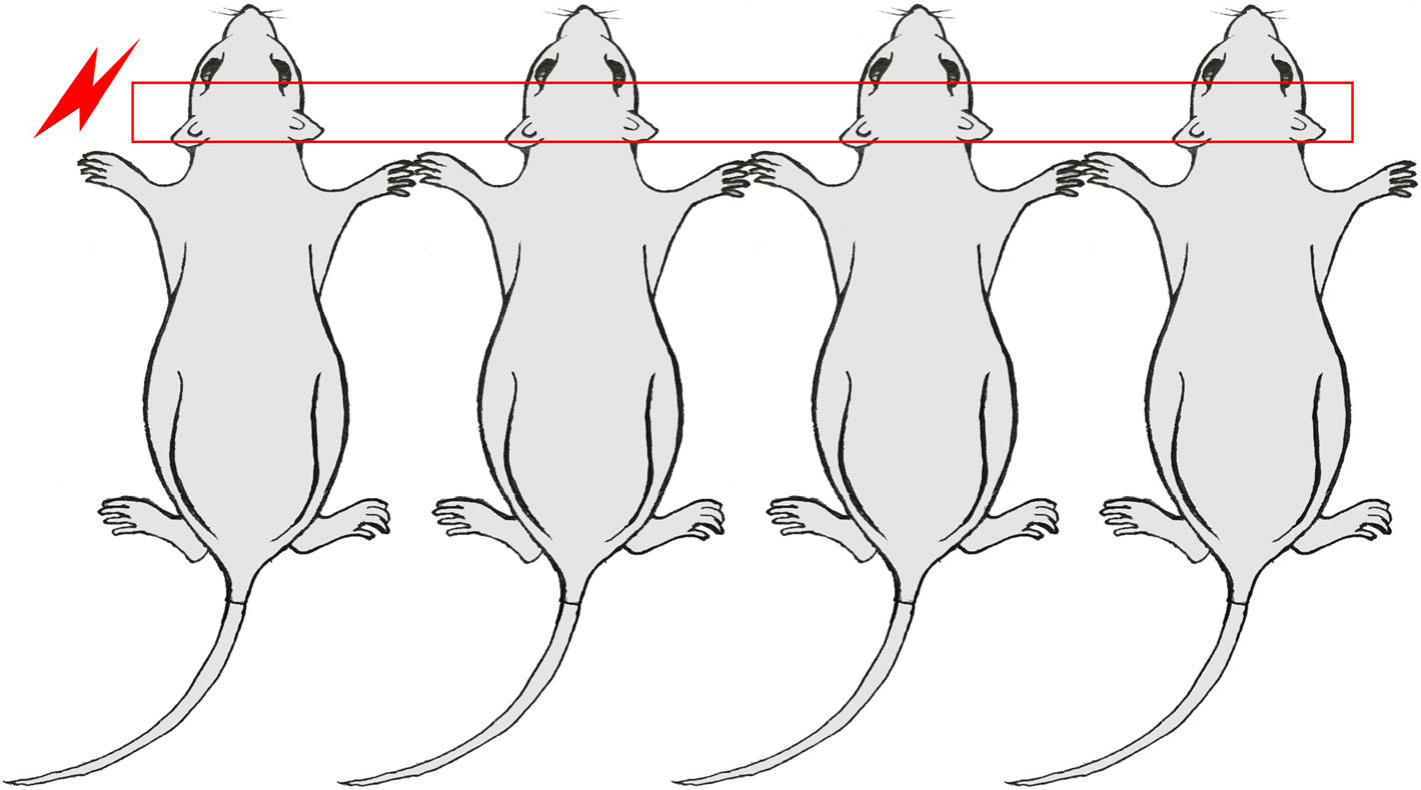
A schematic diagram of the rat brain radiation exposure procedure; the red box represents the exposure area

The rats were anesthetized using intraperitoneal injection of 10% chloral hydrate (0.3 ml/100 g body weight) for cranial irradiation. Anesthetized rats were irradiated using the Varian Clinac iX Medical Linear Accelerator operating at 6 MV photon energy. The dose rate in this setting was 6 Gy/min. For the irradiated rats, 15 Gy and 25 Gy doses were delivered in a single fraction to the two treatment groups. The anterior boundary of the irradiation field was the posterior canthus connection of both eyes, and the posterior boundary was the external auditory canal connection, as shown in Fig. 1. The distance from the radiation source to the surface of the rat’s head was 100 cm. The sham-irradiated controls were anesthetized at the same time points as the rats in the treatment groups but not irradiated.

### In vivo MRI

MRI was performed on a 3 Tesla scanner (Philips Ingenia, Netherlands) equipped with an eight-channel phased-array animal coil (50 mm; Shanghai Chenguang Medical Technologies Co., China) at 4 days and 8 weeks after irradiation. Twenty-four hours before each imaging scan, rats received an intraperitoneal injection of 100 mmol/L MnCl_2_ (60 mg/kg) for contrast enhancement. Standard multislice coronal images were obtained with a 50 × 50 × 16 mm^2^ field of view, a 1.2 mm slice thickness, 200 × 260 matrix, and 12 continuous slices. T1-weighted images were acquired by a T2-weighted turbo spin-echo (TSE) sequence (repetition time (TR) = 510 ms, echo time (TE) = 25 ms, number of signals averaged (NSA) = 12, number of excitations (NEX) = 6, acquisition time = 11 min 01 s). After MEMRI, proton MRS was performed on both hippocampi, and the scanning protocols were as follows: TR/TE =2000/144 ms, voxel of interest (VOI) size = 5 mm × 5 mm × 8 mm, NSA = 256, and acquisition time = 9 min 8 s. The metabolic ratios of N-acetylaspartate (NAA)/creatine (Cr), choline (Cho)/Cr and NAA/Cho were evaluated after the scan.

### Image analysis

**Fig. 2:**
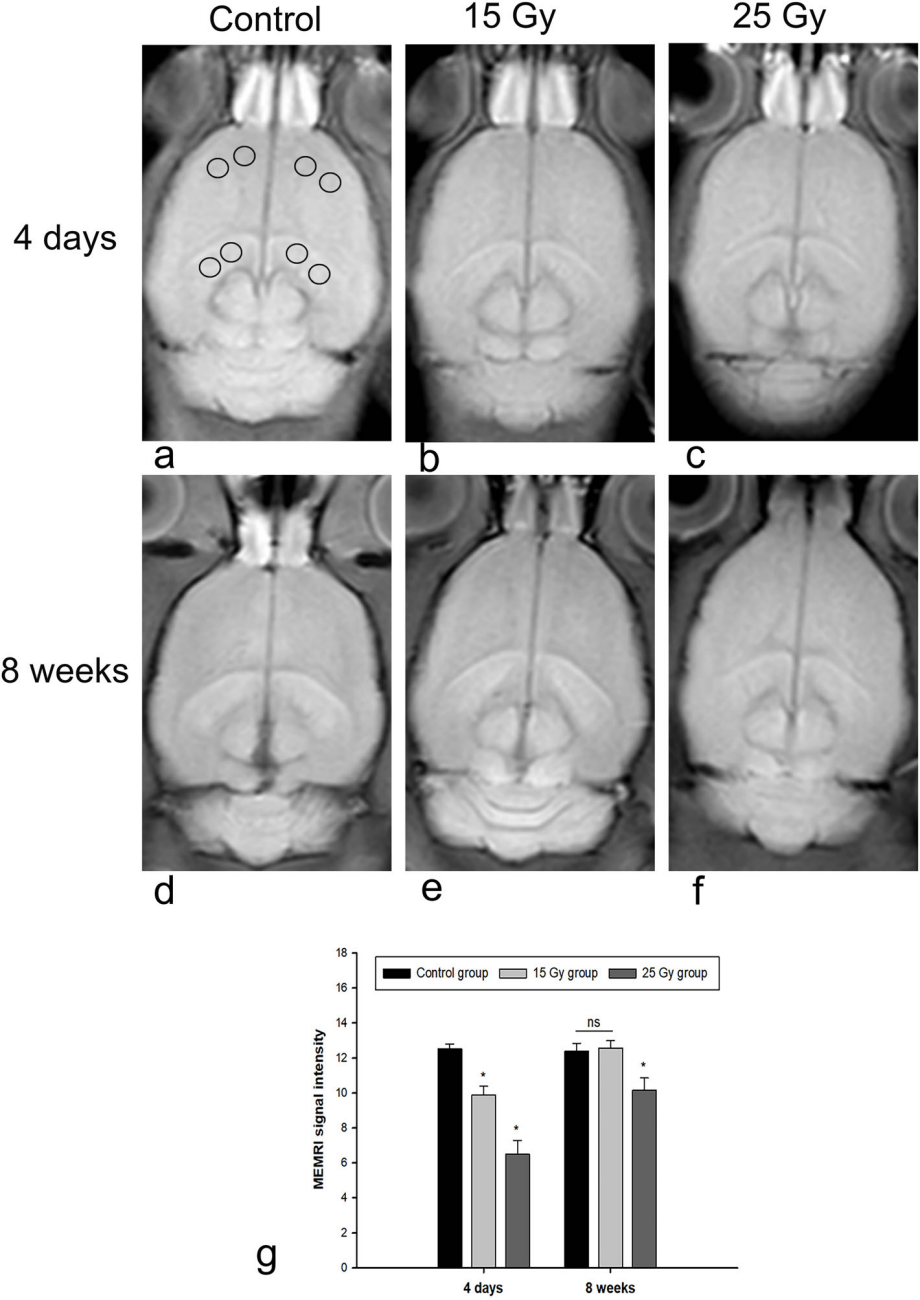
MEMRI SI of the hippocampus after radiation injury. **a-c** show 4 days post irradiation, and **d-e** show 8 weeks post irradiation. a and d show the control group, **b** and **d** show the 15 Gy group, and c and f show the 25 Gy group. The circles in a indicate the method of SI measurement. g shows the quantitative MEMRI SI of the different groups. *P < 0.05, ns, not significant (*P* > 0.05)

All image analyses were performed on a multimodal postprocessing workstation (syngo.via, version VB10B, Siemens, Germany). The mean signal intensity (SI) of MEMRI was calculated in the hippocampus and standardized with the mean signal of the ipsilateral frontal lobe of the brain in the same slice. The normalized enhanced signal was calculated from the following formula: SI = (Sh − Sf)/Sf *100%, where Sh and Sf represent the signal intensities in the region of interest (ROI) in the hippocampus and frontal lobe, respectively. The ROIs are located in the center of each area and have the same size and shape. The SI measurement method is shown in Fig. 2a.

### Western blot analysis and cytokine measurement

After MEMRI, rats were further used for Western blot and cytokine measurements. Fresh hippocampal tissues (*n* = 6 per group) were acquired after rats were euthanized by an overdose of 10% chloral hydrate. Total proteins were extracted using RIPA lysis buffer (Beyotime Biotechnology) and centrifuged (12,000 rpm, 20 min, 4 °C). Supernatants were collected, and proteins were quantified using a bicinchoninic acid (BCA) reagent kit (Beyotime Biotechnology). Equal amounts of protein were resolved by sodium dodecyl sulfate-polyacrylamide gel electrophoresis (SDS-PAGE) on 12% gels, transferred to polyvinylidene difluoride (PVDF) membranes and blocked with 5% fat-free milk at room temperature (20-30 °C) for 1 h. Then, the membranes were incubated with anti-absent in melanoma 2 (AIM2; 1:1000, Abcam, ab180665), anti-cleaved caspase-1 (1:1000, Sigma, SAB4503272-100UG), anti-gasdermin-D (GSDMD; 1:1000, Abcam, ab219800), anti-NLRP3 (1:1000, Abcam, ab214185), anti-NLRP1 (1:1000, Abcam, ab3683), anti-cleaved caspase-3 (1:1000, CST, #9664) and anti-β-actin (1:1000, Abmart, P30002) antibodies overnight at 4 °C. After the membranes were washed with TBST, they were probed with horseradish peroxidase (HRP)-conjugated secondary antibody (1:2000 for anti-rabbit IgG, CST, #7074) at 37 °C for 2 h. Finally, the protein bands were visualized by an enhanced chemiluminescence detection instrument (Tanon 5200). Image analysis was performed using ImageJ software (https://imagej.nih.gov/ij/) to measure the relative density of protein expression.

Hippocampal tissues (*n* = 6 per group) were prepared according to the instructions provided in the enzyme-linked immunosorbent assay (ELISA) kit (Elabscience Biotechnology, China). Briefly, after tissue homogenization and centrifugation, the supernatant was separated for ELISA, and the concentrations of two cytokines, IL-1β (E-EL-R0012c, Elabscience) and IL-18 (E-EL-R0567c, Elabscience), were measured using ELISA kits following the manufacturer’s protocols. The standard curve and regression equation were made according to the standard concentration and optical density value, and the concentration of each cytokine in the sample was calculated according to the regression equation. Data acquisition was performed on SPECTRA MAX190 (Molecular Devices, USA).

### Transmission electron microscopy (TEM) examination

Dorsal hippocampal tissues (mainly including the CA1 area) were fixed using 3% glutaraldehyde followed by 1% osmium tetroxide (*n* = 3 per group). A graded series of acetone (30, 50, 70, 80, 90, 95, and 100%) was used to dehydrate the specimens, which were then embedded in Epon 812. Ultrathin sections (50 nm) were stained with uranium acetate followed by lead citrate. After the sections were stained, they were observed with TEM (H-600IV, Hitachi; or JEM-1400PLUS, JEOL; Japan).

### Statistical analysis

All data are presented as the mean ± standard error of the mean (SEM). Student’s t-test was used for comparisons between two groups. One-way ANOVA was performed to analyze differences among various groups. The data were analyzed with the SPSS 23.0 software package (IBM Corp., Chicago, IL, USA), and statistical significance was defined as *P* < 0.05.

## Results

### Hippocampal inflammasomes and apoptosis levels

**Fig. 3:**
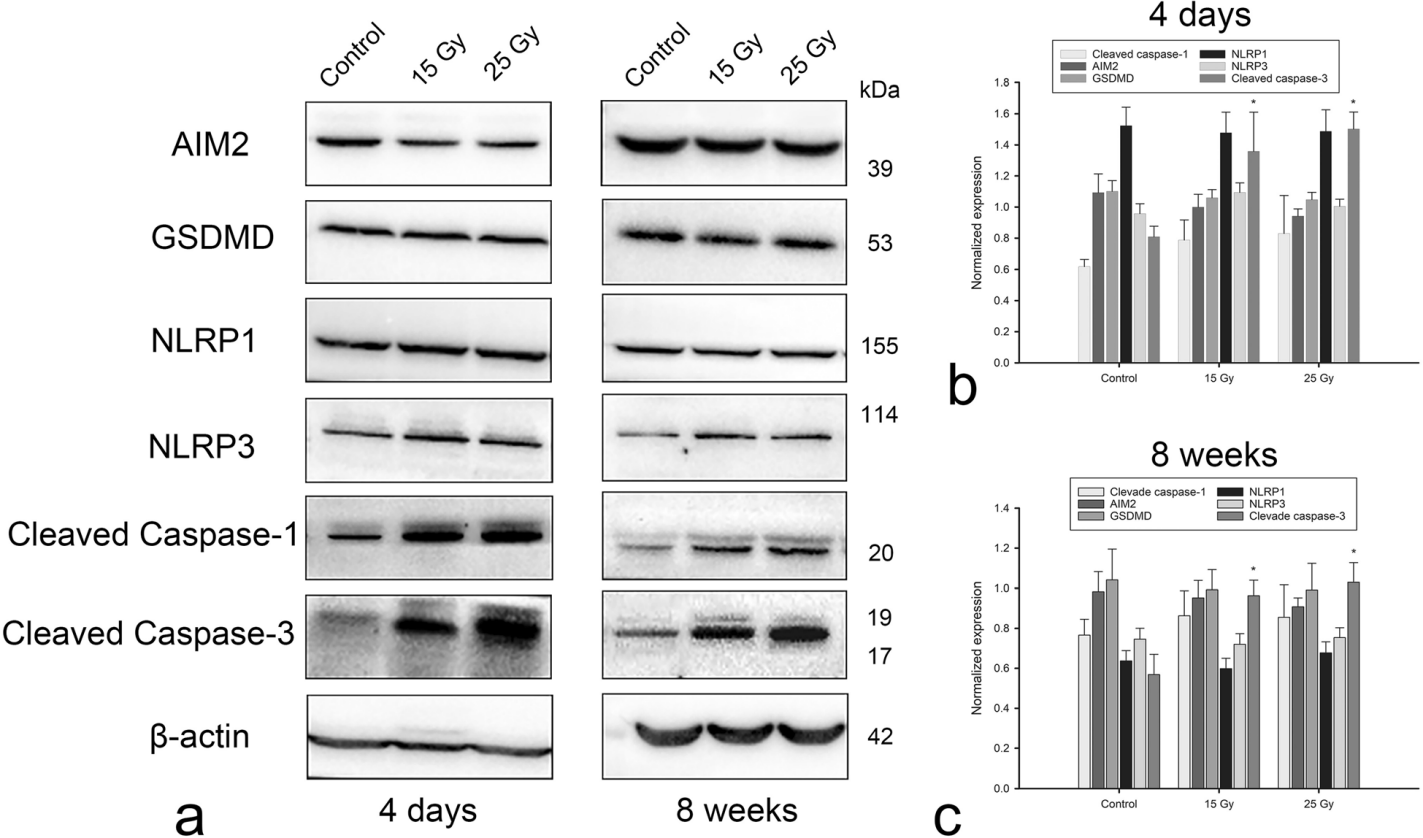
Hippocampal inflammasome expression and apoptosis levels. **a** The expression levels of AIM2, GSDMD, NLRP1 and NLRP3 in the 15 Gy and 25 Gy groups were not significantly different from those in the control group at 4 days or 8 weeks after irradiation. Caspase-1 and caspase-3 levels, especially caspase-3 levels (*P* < 0.05), were higher in the radiation groups than in the control group at 4 days, but these levels began to return to control levels at 8 weeks after irradiation. **b** and **c**. Band intensities were quantified by ImageJ software (b represents 4 days and c represents 8 weeks). *P < 0.05

Four days after irradiation, there was no significant increase or decrease in the hippocampal expression of inflammasome proteins, including AIM2, GSDMD, NLRP1 and NLRP3, in the 15 Gy or 25 Gy groups (all *P* > 0.05). Furthermore, neither treatment group showed a significant change in the expression of these inflammasome proteins 8 weeks after irradiation (all *P* > 0.05) (Fig. 3).

As indicators of apoptosis, cleaved caspase-1 and -3 levels were higher in the irradiation groups than in the control group at 4 days post irradiation, especially for cleaved caspase-3 (*P* < 0.05). Apoptosis in the 25 Gy group was more obvious than in the 15 Gy group. At 8 weeks after irradiation injury, cleaved caspase-1 levels in the 15 Gy and 25 Gy groups were only slightly higher than those in the control group (*P* > 0.05), but cleaved caspase-3 levels in the treatment groups remained significantly higher than those in the control groups (*P* < 0.05) (Fig. 3).

### Hippocampal cytokine levels

**Fig. 4:**
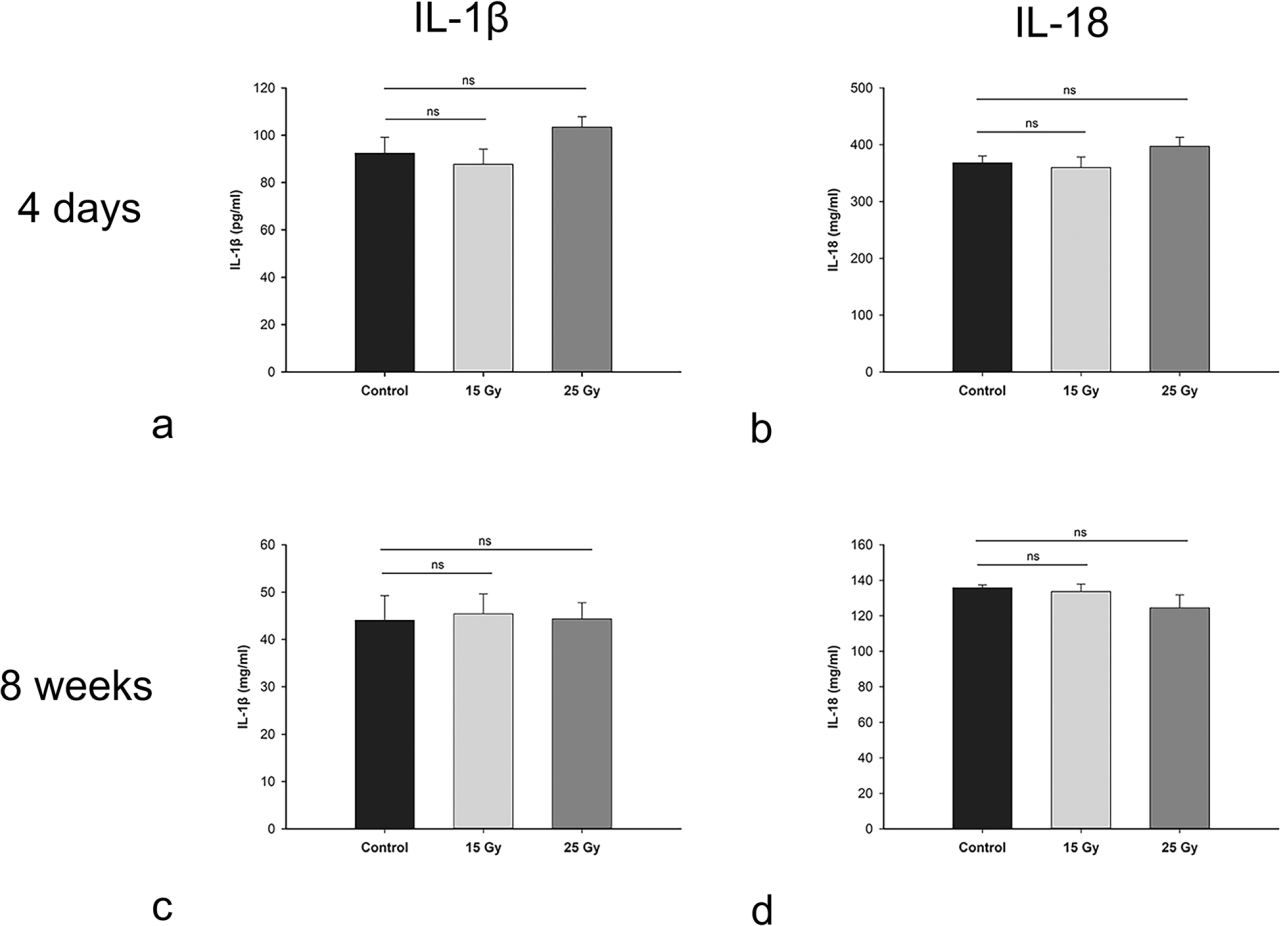
Hippocampal cytokine IL-1β and IL-18 levels. Compared with the control group, the 15 Gy and 25 Gy groups did not show significant increases or decreases in IL-18 or IL-1β at 4 days or 8 weeks after irradiation (all *P* > 0.05). ns, not significant (*P* > 0.05)

The hippocampal levels of two cytokines, IL-1β and IL-18, were measured. This experiment was performed in two stages, not in the same batch. In the acute stage (4 days), there were no significant differences in IL-1β or IL-18 levels among the 15 Gy, 25 Gy and control groups (*P* > 0.05). Furthermore, at 8 weeks post irradiation, there were still no significant differences in IL-1β or IL-18 levels among the groups (*P* > 0.05) (Fig. 4).

### TEM

**Fig. 5:**
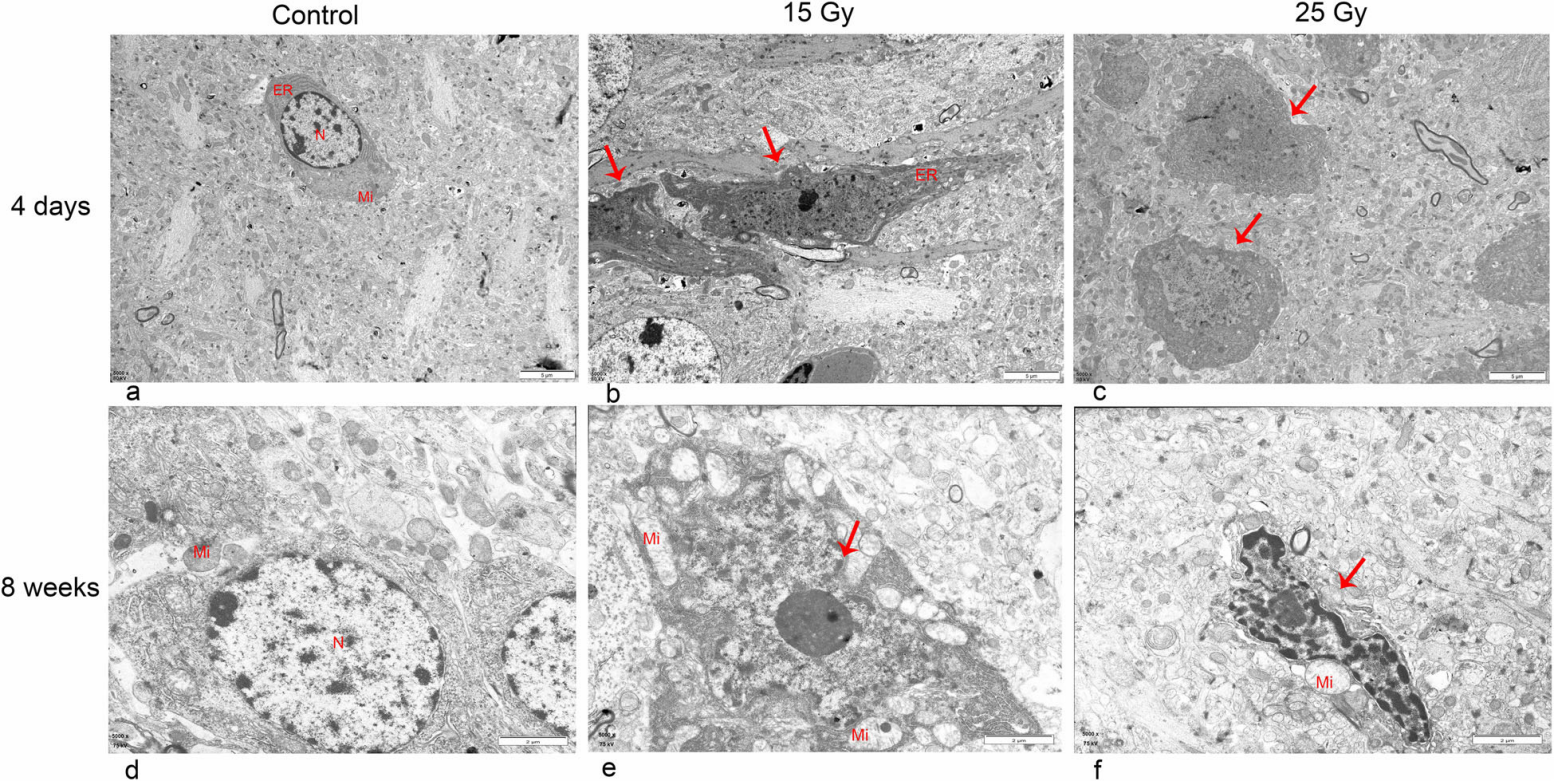
TEM examination of the hippocampus after radiation injury. **a** and **d** show the control group. **b** and **e** show the 15 Gy group, and c and f show the 25 Gy group. After radiation injury, the nuclear chromatin aggregates, the nuclear membrane remains intact, the cytoplasmic electron density increases, mitochondria swell, the crista breaks or even disappears, and the cell volume shrinks. These changes were more severe in the 25 Gy group than in the 15 Gy group and were more pronounced at day 4 (**b** and **c**) than at week 8 (e and f). ER, endoplasmic reticulum, N, nucleus, Mi, mitochondrion. Red arrows indicate apoptotic neurons. Note. **a**-**c** acquired from JEM-1400PLUS, JEOL, d-f acquired from H-600IV, Hitachi

TEM revealed that radiation injury reduced the cell volume, aggregated the nuclear chromatin, increased the electron density of the cytoplasm, caused the mitochondria to swell, and broke down or destroyed the crista but left the cell membrane and nuclear membrane intact. The number of apoptotic neurons in the 25 Gy group was greater than that in the 15 Gy group both at 4 days and 8 weeks after irradiation. For the 25 Gy group, apoptosis was more obvious on day 4 than at 8 weeks, as shown in Fig. 5.

### MEMRI

There was no significant difference in SI between the left hippocampus and the right hippocampus in any of the groups (all *P* > 0.05). The SI of both the 15 Gy and 25 Gy groups was lower than that of the control group at 4 days but began to return to control levels at 8 weeks after irradiation. Four days after irradiation, the 25 Gy group exhibited a significantly lower SI than the control group (*P* < 0.05). At 8 weeks after irradiation injury, the SI of the 15 Gy group and the 25 Gy group began to recover to different degrees; the increase in the 15 Gy group was more obvious than that in the 25 Gy group, and there was no significant difference in SI between the 15 Gy group and control group at 8 weeks (*P* > 0.05). However, the SI of the 25 Gy group was still significantly lower than that of the control group at 8 weeks after irradiation injury (*P* < 0.05) (Fig. 2, *n* = 9 per group).

### MRS

**Fig. 6:**
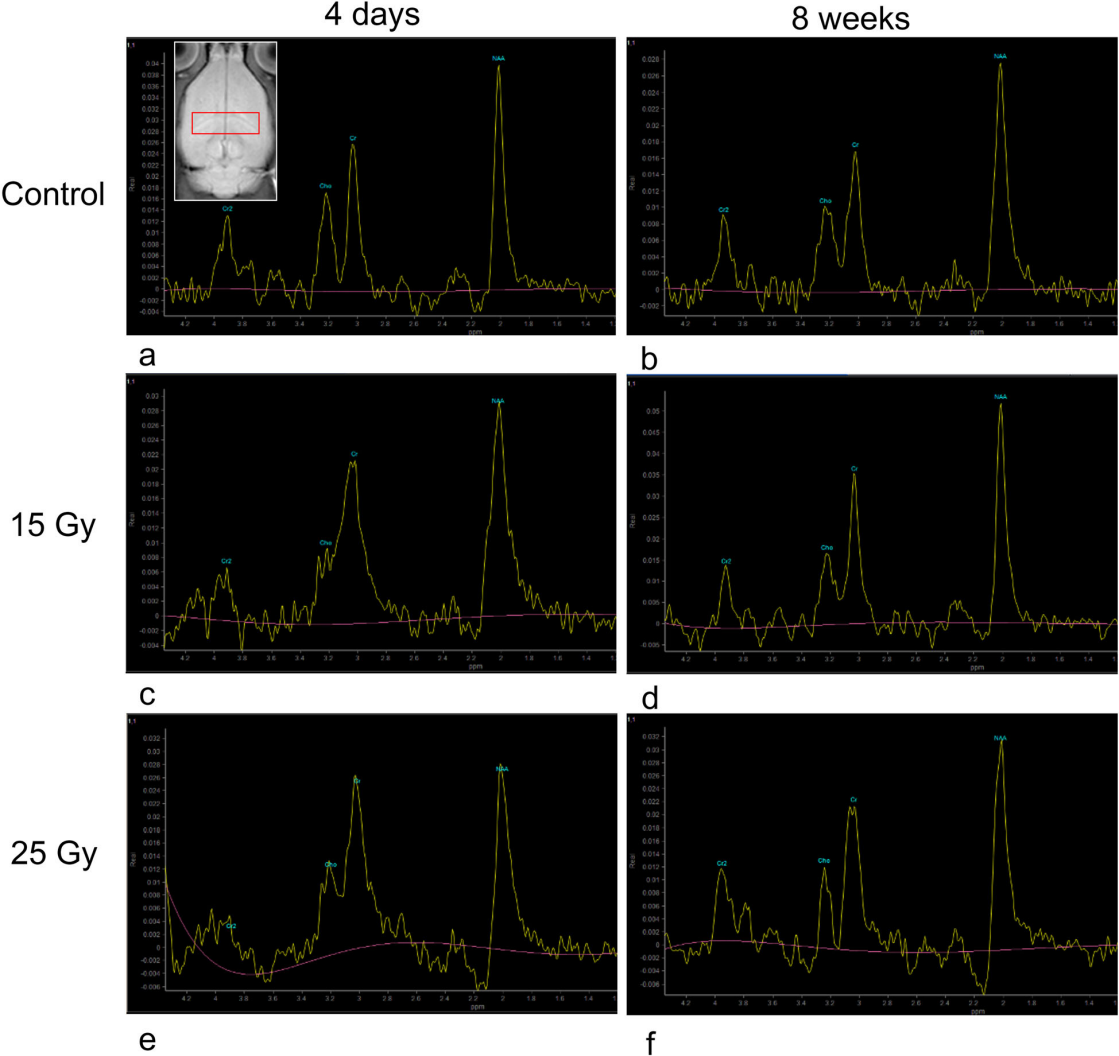
MRS of the rat hippocampus after radiation injury. **a** demonstrates the location of the ROI (red rectangular box) in hippocampus. **b**-**g** show the MRS of the different groups. The NAA/Cr ratio in the 15 Gy group and 25 Gy group was significantly lower than that in the control group on the 4th day after radiation injury, but the ratios at 8 weeks were partially recovered

The metabolite ratios measured in each group are presented in Table 1. On day 4 post irradiation, compared to the ratio in the control group, the metabolic NAA/Cr ratio in both the 15 Gy group and the 25 Gy group was significantly lower (*P* < 0.05). At 8 weeks, the NAA/Cr ratio in the 15 Gy group showed significant recovery to levels not significantly different from that in the control group (*P* > 0.05), but the NAA/Cr ratio in the 25 Gy group remained significantly lower than that in the control group (*P* < 0.05). Significant reductions in the metabolic Cho/Cr ratio were observed in the 15 Gy group and 25 Gy group at day 4 (compared with control *P* < 0.05). The NAA/Cho ratio in the 25 Gy group was significantly higher than that in the control group at 4 days post irradiation (*P* < 0.05). (Fig. 6).

**Table 1:** Metabolite ratios of rat hippocampus in irradiated and control groups

Group	NAA/Cr	Cho/Cr	NAA/Cho
Control group	1.3711 ± .04638	.7944 ± .04616	1.7578 ± .08388*
15 Gy 4 days	1.1412 ± .07006#	.5513 ± .05269#	2.1413 ± .14321
25 Gy 4 days	1.1014 ± .06874#	.4843 ± .07767#	2.9271 ± .53783*
P	*P* < 0.05 (#*P* > 0.05)	*P* < 0.05 (#*P* > 0.05)	*P < 0.05
Control group	1.3900 ± .02147*	.6089 ± .05524	2.3678 ± .29411
15 Gy 8 weeks	1.2533 ± .07476	.5233 ± .05050	2.5567 ± .22618
25 Gy 8 weeks	1.1667 ± .04113*	.5656 ± .07871	2.7133 ± .50107
P	*P < 0.05	*P* > 0.05	*P* > 0.05

## Discussion

The present study demonstrates that inflammasomes in the hippocampus were not significantly upregulated or downregulated after RIBI, and pyroptosis was not observed; however, apoptosis was activated, especially in the acute phase. These results suggest that apoptosis, not inflammasomes or pyroptosis, plays a major role at the acute and early-delay stages of RIBI. These changes in the hippocampus can be observed by MEMRI and MRS.

Inflammasomes are mainly divided into the classical pathway, which is dependent on caspase-1, and the nonclassical pathway, which is dependent on caspase-4/5/11 [27]. The classical inflammasomes that depend on caspase-1 include NLRP1, NLRC4, NLRP3, AIM2 and pyrin, the activation of which is mainly through NLR-dependent activation signals and is followed by the recruitment of caspase-1 through the adaptor protein [28]. The active form of caspase-1 cleaves GSDMD, allowing GSDMD to release the active N-terminal protein. The N-terminal protein binds to the cell membrane and oligomerizes on the cell membrane to form a pore-like structure, which releases the cell contents and causes pyroptosis [29]. In this study, we measured changes in inflammasome protein expression in juvenile rats at different time points after different radiation doses. We demonstrated that exposure to cranial irradiation with 15 Gy and 25 Gy did not result in significant changes in inflammasomes at either 4 days or 8 weeks. Our findings are inconsistent with those of previous studies and may be related to the observed time points and radiation dose. Some studies have shown that increased expression of inflammasomes and pyroptosis can be observed after a low dose (< 10 Gy) of radiation and in the hyperacute stage (< 24 h) [13, 30]. However, Liao et al. demonstrated that mouse brains exposed to 15 Gy radiation experienced significant activation of the NLRP3 inflammasome and caspase-1 in cortices at 1 month post irradiation [17]. These different results may be related to differences in the radiation response between animal species and differences in parts of the brain examined. In vitro experiments by Liu et al. showed that 10 Gy and 20 Gy radiation increased caspase-1 activation in bone marrow-derived macrophages at 3 h after irradiation in a dose-dependent manner [31]. These authors also showed that 10 Gy radiation in vivo induced pyroptosis and caspase-1 activation. We also found that the main mechanism of RIBI in the early-delayed stage did not include activation of inflammasomes or pyroptosis. This finding is helpful for the rational choice of treatment options at different stages.

Although we did not observe radiation-induced pyroptosis, we did observe apoptosis and found that these changes could be imaged by MEMRI. Proliferating cells are more susceptible to radiation in juveniles. We found that the amount of apoptosis in the hippocampus and the degree of caspase-1/3 activation after acute-phase irradiation were dose dependent. However, the degree of caspase-1 activation decreased after the acute phase, whereas caspase-3 activation remained significant at 8 weeks. These data suggest that apoptosis persisted in the early-delayed stage, which was also confirmed by TEM. TEM revealed that apoptosis was more obvious in the acute stage and after high-dose irradiation. Under the conditions examined, hippocampal apoptosis rather than pyroptosis occurred in the acute stage. The occurrence of apoptosis or pyroptosis after radiation injury may be related to radiation dose, fractionation and time intervals. Although the levels of proinflammatory cytokines IL-1β and IL-18 were not significantly higher than control levels in the early stage of this study, previous reports have shown increases in other inflammatory mediators, such as T cells, CD11c-positive cells, tumor necrosis factor (TNF)-α and intercellular adhesion molecule (ICAM)-1, in this stage [32–34]. In this study, we mainly sought to examine the occurrence of pyroptosis and did not study other proinflammatory cytokines.

Given the limitations of the clinical MRI device used in this study, the effects of a low radiation dose may not be detectable due to the insufficient sensitivity of the device. Thus, in designing our study, we selected one high and one low dose to increase our chances of producing observable biological effects while also avoiding excessive mortality. Although doses lower than 15 Gy can cause physiological changes, the changes in the MR image are slight. However, 25 Gy whole-brain radiotherapy has been shown to cause marked impairment in rats [35] and damage that is very close to the serious radiation damage observed in humans. We examined the effects of 25 Gy in this study because the damage caused by this dose of whole-brain radiotherapy is sufficient to result in changes in MRI.

In vivo MEMRI can be used to detect changes associated with brain injury induced by radiation. A previous study using a prenatal radiation exposure model demonstrated that changes to MEMRI were probably dominated by decreases in cell number and excessive increases in cell apoptosis [36]. In this study, we observed a significant decrease in hippocampal SI after irradiation, which may be due to apoptosis. Similarly, Saito et al. [37] observed that MEMRI signals in the CA1/2 regions of the hippocampus disappeared after prenatal X-ray irradiation. Apoptosis was related to irradiation dose, with the higher dose associated with more obvious apoptosis and lower MEMRI signals. The T1 relaxation enhancement of MEMRI is in direct proportion to the ion concentration of Mn^2+^ entering the cell through Ca^2+^ channels [38]. Apoptosis leads to a decrease in Mn^2+^ uptake, which in turn leads to a decrease in enhancement. Thus, MEMRI can reflect the apoptosis of hippocampal cells after radiation injury. Notably, MEMRI has revealed some differences in the manifestations of apoptosis caused by RIBI and hypoxic-ischemic encephalopathy (HIE). HIE often leads to severe damage, with neuronal death occurring in the early acute phase and resulting in a decrease in MEMRI SI. However, at later time points, an increase in SI is caused by a large number of activated microglia and inflammation around the lesion areas [39–41].

In addition, we observed a significant decrease in hippocampal T1 relaxation enhancement in the acute phase (4 days), which gradually recovered in the early-delayed phase (8 weeks) but was still lower than the normal level. This pattern suggests a decrease in apoptosis after the acute phase, which was confirmed by Western blot results (caspase-3 decreased to near normal levels). At the same time, there may be some spontaneous repair, but this repair is very limited and cannot return the hippocampus to the normal state, especially after high-dose irradiation. Another possible reason for the increase in the MEMRI signal and the decrease in apoptosis at 8 weeks is that some nerve cells may continue to develop, and some functions may be restored after radiation injury in young rats. However, fully recovering from damage caused by high-dose irradiation is difficult.

The present 1H MRS study revealed significant changes in hippocampal metabolites post irradiation. The metabolite NAA is a marker of neuronal density and function. Neuronal damage, apoptosis, and dysfunction after radiation injury result in a decrease in NAA [42, 43]. Cr is a marker of energy metabolism and is usually quite stable [44]. Therefore, the ratio of metabolites to Cr is commonly used to determine changes in metabolites in individuals. We observed a significantly lower NAA/Cr ratio on day 4 in both the 15 Gy and 25 Gy groups, especially in the 25 Gy group, than in the control group. At 8 weeks, the NAA/Cr ratios in the 15 Gy and 25 Gy groups began to return to control levels, but the ratio in the 25 Gy group remained significantly lower than that in the control group. The changes in the NAA/Cr ratio were consistent with the changes in apoptosis observed in this experiment. Thus, changes in the NAA/Cr ratio can also indicate apoptosis of cells after RIBI. In addition, the Cho/Cr ratio decreased to different degrees after radiation in both groups.

Both MEMRI and MRS have been shown to be very promising methods for evaluating hippocampal damage, which is important to prevent during radiation therapy. A variety of measures have been attempted to minimize the radiation dose to the hippocampus in clinical trials. A phase III clinical trial (NRG Oncology CC001, NCT02360215) showed that combined treatment with hippocampal avoidance whole-brain radiotherapy (HA-WBRT) and memantine was better than combined treatment with WBRT plus memantine in preserving cognitive function and patient-reported symptoms in patients with brain metastases [45]. The second ongoing phase III trial, NRG Oncology-CC003 (NCT02635009), is assessing the effects of WBRT with or without HA-WBRT in treating patients with limited-stage or extensive-stage small-cell lung cancer. In addition, because radiation may increase antigen presentation and promote the abscopal effect, several clinical trials (NCT02648633, STERIMGLI -NCT02866747, etc.) are underway to examine the efficacy of checkpoint inhibitors in combination with radiation therapy in recurrent glioblastoma [46]. We expect these trials of combination therapy to show synergistic effects, thereby minimizing the brain radiation dose and reducing adverse effects.

Several limitations of this study should be noted. First, we did not use a lower dose of radiation or observe the effects at an earlier time point, which may be why we did not find a change in inflammasomes. However, our study indicated that inflammasome activation and pyroptosis may be related to radiation dose and observation timepoint but are not long-term consequences. Second, we did not perform a comparative study on different subregions of the hippocampus. Different subregions may have different radiosensitivity and different outcomes. In the future, using ultrahigh magnetic field MRI, such as 7 T or higher, may be a better strategy to study the effects of clinical doses and examine different subregions in the hippocampus. Third, we only studied changes in the acute phase and early-delayed phase but not in the chronic stage, and the changes in the chronic stage may be different.

## Conclusion

This study found that apoptosis, but not inflammasome activation or pyroptosis, plays a major role in the acute and early-delayed phases after RIBI at specific doses and that cell apoptosis can be detected by MEMRI. MRS is an important supplement to MEMRI, and the combination of the two can better evaluate changes in apoptosis after radiation injury.

## Data Availability

The datasets used and/or analyzed during the current study are available from the corresponding author on reasonable request.
